# Umbilical Cord MSCs and Their Secretome in the Therapy of Arthritic Diseases: A Research and Industrial Perspective

**DOI:** 10.3390/cells9061343

**Published:** 2020-05-28

**Authors:** Chiara Arrigoni, Daniele D’Arrigo, Valeria Rossella, Christian Candrian, Veronica Albertini, Matteo Moretti

**Affiliations:** 1 Regenerative Medicine Technologies Laboratory, Ente Ospedaliero Cantonale (EOC), via Tesserete 46, 6900 Lugano, Switzerland; chiara.arrigoni@eoc.ch (C.A.); daniele.darrigo@eoc.ch (D.D.); 2 Swiss Stem Cells Biotech, Via Pizzamiglio 12, 6833 Vacallo, Switzerland; valeria.rossella@stembiotech.ch (V.R.); veronica.albertini@stembiotech.ch (V.A.); 3 Unità di Ortopedia e Traumatologia, Ospedale Regionale di Lugano, Ente Ospedaliero Cantonale (EOC), via Tesserete 46, 6900 Lugano, Switzerland; christian.candrian@eoc.ch; 4 Faculty of Biomedical Sciences, Università della Svizzera Italiana, Via Buffi 13, 6900 Lugano, Switzerland; 5 Cell and Tissue Engineering Laboratory, IRCCS Istituto Ortopedico Galeazzi, via R. Galeazzi 4., 20161 Milano, Italy

**Keywords:** umbilical cord MSC, secretome, osteoarthritis, extracellular vesicles, cell therapies

## Abstract

The prevalence of arthritic diseases is increasing in developed countries, but effective treatments are currently lacking. The injection of mesenchymal stem cells (MSCs) represents a promising approach to counteract the degenerative and inflammatory environment characterizing those pathologies, such as osteoarthritis (OA). However, the majority of clinical approaches based on MSCs are used within an autologous paradigm, with important limitations. For this reason, allogeneic MSCs isolated from cord blood (cbMSCs) and Wharton’s jelly (wjMSCs) gained increasing interest, demonstrating promising results in this field. Moreover, recent evidences shows that MSCs beneficial effects can be related to their secretome rather than to the presence of cells themselves. Among the trophic factors secreted by MSCs, extracellular vesicles (EVs) are emerging as a promising candidate for the treatment of arthritic joints. In the present review, the application of umbilical cord MSCs and their secretome as innovative therapeutic approaches in the treatment of arthritic joints will be examined. With the prospective of routine clinical applications, umbilical cord MSCs and EVs will be discussed also within an industrial and regulatory perspective.

## 1. Introduction

Arthritic diseases include different pathologies, such as rheumatoid arthritis (RA), a chronic inflammatory disorder mainly driven by autoimmune reactions. Genetic predisposition is at the basis of its development, while other genetic and environmental cues contribute to its clinical onset, characterized by a proinflammatory and degenerative synovial response, inducing joint inflammation, pain and disability [1]. Osteoarthritis (OA), the most common arthritic disease, is a degenerative joint disease causing a progressive degradation of articular cartilage and subchondral bone [2], both leading to a significant loss of joint function, heavily affecting the patient’s quality of life. OA is characterized by a multifactorial etiology, including idiopathic, genetic, metabolic, inflammatory factors and joint traumas. All these predisposing factors lead to the establishment of a positive proinflammatory feedback among articular cells, associated to chondrocytes metabolic imbalance and ultimately causing the progressive degradation of the cartilaginous matrix [3]. RA prevalence is estimated around 1% globally and is mainly related to the presence of specific genetic risk factors [1]. OA prevalence is instead increasing in developed countries, due to population aging and to the promotion of an active lifestyle at all ages [4]. It is estimated that approximately 240 million people worldwide are affected by OA, corresponding to a percentage of around 10% of men and 18% of women above 60 years [5]. This disease also represents a huge economic cost for healthcare systems, exceeding 200 million €/year in Europe [6]. Current therapeutic options are predominately palliative and still far from halting disease progression [7], leaving the only final option of invasive surgery (arthroplasty/osteotomy). For this reason, research is focusing on the development of new treatments for the healing of diseased joint tissues [6]. Recently, it has been evidenced the key role of inflammation in the insurgence of OA, shifting the classification of OA from a purely degenerative disease to an inflammation-driven condition [8]. Accumulating evidences point out that synovitis, with the associated production of inflammatory mediators, can be recognized as a key OA driver, and thus, targeting the inflammatory response represents an appealing therapeutic strategy [6].

In this scenario, different approaches have been proposed, including injections of biological molecules such as hyaluronic acid (HA) and platelet-rich plasma (PRP). Recent meta-analyses highlighted how the injection of HA is a safe procedure but without evidence of efficacy in slowing OA progression [6], and thus, no clear indications for its use in OA are present [9]. Contrasting evidence is reported also for the use of PRP, whereby a superior effect on pain relief as compared to HA injections has been assessed [10], although a significant placebo effect has been associated to its use [11].

To overcome the limitations of these injective preparations, the injection of cells capable of engrafting in the damaged cartilage and promoting its healing, such as autologous chondrocytes, has been proposed [6]. However, despite initial promising results, poor functionality and quality of the synthesized extracellular matrix (ECM) have been reported, leading to a limited efficacy in patients older than 40 years [12]. As an alternative, the use of progenitor cells such as mesenchymal stromal cells (MSCs) from various sources has been attempted but with questionable outcomes on cartilage regeneration [6]. MSCs, are self-renewable multipotent cells that have been isolated from different neonatal and adult tissues. They are endowed with several features that make them attractive for cell therapy, including easy in vitro handling, genomic stability, few ethical issues and the differentiation ability towards all the three lineages [13]. The rationale behind the use of MSCs for cartilage repair has, in the past, been based on their ability to differentiate into chondrocytes and replace injured cartilage [14]. However, increasing evidence suggests that MSCs’ contribution may lie in orchestrating the regenerative process also through the secretion of a wide range of trophic factors that modulate the injured tissue environment [15]. Thus, several groups are now focused on establishing the potential of MSCs’ secretome and, in particular, of their secreted extracellular vesicles (EVs) as a therapy for OA joints [16]. EVs are nano-sized lipid vesicles secreted by almost all the cell types, playing a fundamental role in the cell-cell communication process. Their cargo consists of several different biologically active compounds, such as proteins, enzymes and nucleic acids, that can regulate the behavior of the target cells [17].

MSCs are generally used within an autologous paradigm; i.e., they are harvested from the patient himself [18], with drawbacks related to the need for a further intervention and to significant hurdles in standardization and quality control strategies. A possible solution resides in the use of allogeneic MSCs, such as those isolated from cord blood (cbMSCs) and Wharton’s jelly (wjMSCs), which are characterized by a low immunogenicity and demonstrated promising results for the cure of several diseases, including spinal cord injuries, liver fibrosis and heart failure [19]. These two MSCs populations are isolated from different compartments of the umbilical cord: cbMSCs are harvested from the blood contained in the umbilical cord, while wjMSCs are isolated from the connective tissue (called Wharton’s jelly) that surrounds its blood vessels. When cultured in vitro, cbMSCs and wjMSCs display a similar immunophenotype, fibroblast-like spindle morphology and express the same surface marker [20].

In this review, we will discuss the application of MSCs from the umbilical cord and of their secretome as possible therapeutic approaches against arthritic diseases. Beyond critically discussing the results of investigations in vitro, in vivo and in clinical settings, we will also give an industrial perspective on the advantages and bottlenecks related to both therapeutic options. Literature search was performed, including the studies published until early 2020 and that regarded MSCs isolated from the umbilical cord and their secretome and EVs, employed in vitro, in vivo and in clinical settings for the treatment of OA and RA. In particular, we used the following keywords: mesenchymal stem/stromal cells, MSC, exosome, (micro)vesicle, ectosome, secretome, associated with umbilical cord, Wharton’s jelly, cord blood and cartilage, Synovi*, chondrocyte, chondral, osteoarthritis, OA, rheumatoid arthritis and RA. From the studies found, we excluded nonoriginal articles, studies not related to OA or RA, tissue-engineering approaches and articles not written in the English language.

## 2. Features of MSCs from the Umbilical Cord

In the last thirty years, MSCs have been isolated from different sources and, most frequently, from the bone marrow. First reports on the identification of umbilical cord MSCs categorize them as fibroblast-like morphology cells, with a mesodermal trilineage potential to differentiate into bone; cartilage; fat and presenting a characteristic MSC immunophenotype (CD44, CD73, CD90 and CD105 positive and CD14, CD19, CD31, CD34, CD45 and HLA-DR negative). Successively, many other studies described stromal cells isolated from umbilical cord-derived Wharton’s jelly connective tissue with characteristics highly similar to MSCs isolated from the bone marrow [21,22]. In the definition of “umbilical cord MSCs”, both cbMSCs and wjMSCs can be included. Despite the different origins of the tissues, both cell types are considered multipotent [23,24].

Different studies extensively explained how difficult the stromal cell isolation from the cord blood is, raising some doubts on the possibility of harvesting MSCs from this source [25,26,27]. Conversely, it seems much easier to extract MSC-like cells from Wharton’s jelly connective tissue also in terms of the cell isolation yield [26].

Nevertheless, wjMSCs seem to have a lower differentiative potential into bone cells, adipocytes and chondrocytes compared to cbMSCs and bone marrow MSCs (bmMSCs). In support of this evidence, when wjMSCs are subcutaneously transplanted into immunocompromised mice, they are not able to form heterotopic ossicles in vivo [28]. However, in view of using umbilical cord MSCs as a therapy for OA treatment, it is particularly interesting to investigate their chondrogenic potential as a promising alternative cell source for cartilage repair. In this context, cbMSCs can be easily induced to differentiate into chondrocytes [29,30], showing a higher chondrogenic differentiation potential as compared to bmMSCs [31] and adipose-derived MSCs (atMSCs) [32]. wjMSCs are less chondrogenic than cbMSCs [33,34], forming less hyaline cartilage tissue [35]. However, wjMSCs are able to biosynthesize collagen I and glycosaminoglycans under proper in vitro culture conditions, thus remaining promising candidates for future tissue engineering applications such as the treatment of temporomandibular joint (TMJ) disorders [36].

In view of a potential therapeutic application of wjMSCs as an allogeneic source, it is crucial to assess their behavior when in contact with host immune cells. In the literature, it has been shown that wjMSCs possess immunosuppressive properties even more evident than those of bmMSCs [37], suppressing the proliferation of stimulated immune cells in vitro. Furthermore, wjMSCs show low immunogenicity, which renders them suitable for allogeneic transplant [38], avoiding rejection in vivo and maintaining their properties even after chondrogenic differentiation [39].

## 3. Cord Blood and Wharton’s Jelly MSCs for OA and RA Therapy

Thanks to their chondrogenic potential and immunomodulatory features, wjMSCs and cbMSCs have been regarded as potential therapeutic agents against arthritic diseases due to the key role of inflammatory processes and associated joint cartilage degradation. In particular, early evidences that emerged from in vitro studies on cell cultures (summarized in Table 1) has been confirmed in different animal models, from mice to horses (summarized in Table 2), finally leading to some recently reported clinical trials (summarized in Table 3).

Preliminary in vitro results on cocultures between wjMSCs and ILβ1-stimulated chondrocytes showed reduced expressions of cartilage-degrading enzymes, such as MMP-1 and MMP-3, as compared to ILβ1-stimulated chondrocytes alone [40]. The same decrease was observed also in OA chondrocytes after cocultures with wjMSCs [41]. Taken together, these results suggest a protective effect of wjMSCs on damaged chondrocytes and have been confirmed in vivo, whereby the injection of wjMSCs in rat models of OA showed reduced secretions of cartilage-degrading enzymes [42], inflammatory cytokines [43], increased cartilage matrix production and decreased chondrocyte apoptosis [44]. Beside their direct effects on cartilage cells, wjMSCs act also on other articular cells, such as synovial fibroblasts, playing a key role in synovitis. Indeed, synovial fibroblasts from either OA or RA patients cocultured with wjMSCs showed a reduced expression of inflammatory molecules and matrix-degrading enzymes as compared to synovial fibroblasts in control conditions, associated with a higher production of cartilage matrix proteins collagen II and aggrecan in fibroblasts [45,46,47]. These results have been confirmed also in a horse model of synovitis, in which the injection of allogeneic cbMSCs led to a decrease in the number of immune cells in the synovial fluid as compared to the control, showing the possibility to reduce inflammation and associated immune cell recruitment [48].

The therapeutic effects of wjMSCs have been explored in other large animal models such as dogs [49], showing an improvement of OA disease hallmarks, detected with clinical imaging evaluations (MRI, ecography and Rx), analyses of harvested cartilage surfaces and the decrease of inflammatory cytokines [49]. Interestingly, wjMSCs retained their immunomodulatory and cartilage protective effects also in a xenogeneic transplantation scenario. Indeed, human wjMSCs injected into a mini-pig model reduced histological OA signs, increasing cartilage matrix production [50]. Furthermore, horse-derived wjMSCs were able to modulate the synovial inflammatory response in rabbits, especially when injected early after cartilage damage induction, suggesting that wjMSCs secrete bioactive molecules that work even across barrier species, modulating the expression of genes related to inflammation and matrix turnover in synoviocytes [51]. When compared to other sources of MSCs, like the bone marrow, cbMSCs demonstrated a superior secretion of anti-inflammatory cytokines (IL-10 and IL-6) and a higher capacity to restore cartilage matrix production in a 3D cartilage construct [52]. This result has been confirmed in a more recent study showing that the injection of allogeneic cbMSCs in horses led to a lower inflammatory response and reduced cartilage degradation as compared to allogeneic and even autologous bmMSCs [53].

Summarizing, evidence from preliminary studies supports the anti-inflammatory potential of wjMSCs in arthritic diseases, associated with a higher production of anti-inflammatory molecules. Furthermore, they can promote cartilage regeneration through the stimulation of matrix production by diseased chondrocytes and synoviocytes.

Following promising in vitro and in vivo results, a first clinical application has been attempted using wjMSCs included in a hydrogel matrix in a patient with an untreatable osteochondral defect, showing safety and the improvement of symptoms at a long-term follow-up (five years) [54]. The same group reported a wider phase I/II single arm study in which seven patients with severe OA (Kellgren-Lawrence (K-L) grade 3 and International Cartilage Repair Society (ICRS) grade 4) were treated with the same hydrogel matrix embedded with wjMSCs. No serious adverse events were encountered, and biopsies performed after the first year showed cartilage regeneration, confirmed two years later by MRI analyses [55]. Positive results of this technique for the treatment of severe OA have been recently confirmed in a retrospective case-series on a further 128 patients [56]. Despite the encouraging findings, cells were administered through an invasive surgical procedure riskier for the patients as compared to an intra-articular injection. In this view, a group from Chile recently published the results of a controlled randomized phase I/II trial comparing repeated doses of wjMSCs with a single cell dose and with double HA injections in a group of 29 patients with mid-grade OA (K-L grades II-III). At the short term, the main side effects of the wjMSCs injections were acute synovitis and joint effusion; however, at 12 months of follow-up, the group receiving double MSC injections had significantly reduced pain and improved function as compared to both single MSC doses and double HA injections [57]. The positive effects on pain reduction and clinical scores with the injection of wjMSCs were confirmed by a recent single-arm study on 29 patients with mild-to-severe OA [58].

In conclusion, recently published clinical trials demonstrated the safety and efficacy of MSCs from Wharton’s jelly in the treatment of OA, although follow-up periods are still short, and the technique for cell implantation has still to be optimized.

## 4. Secretome of Cord Blood and Wharton’s Jelly MSC for OA and RA Therapy

As previously described, MSCs from the umbilical cord demonstrated huge potential when being used in cell-based regenerative therapies compared to other MSCs sources. However, the use of these cells in clinical practice is still hampered by some limitations, such as the maintenance of biological activity, and the identity and the amount of the bioactive factors, as well as logistics issues [64]. Therefore, efforts are underway in finding a cell-free method that maintains the same biological effects of MSCs, resulting in an increased interest on their secretome and small-size EVs or exosomes. Indeed, it has been reported that these cell products could retain all the advantages of the parental cells, such as low immunogenicity, immunoregulation properties and the delivery of bioactive factors [64,65,66].

Umbilical cord MSCs’ secretome appears to be significantly different from the secretome of amniotic membrane-derived [67,68], adult adipose [67,69] and adult bone marrow-derived MSCs [68,69]. These differences involve a different presence of angiogenic factors [68], a lower amount of metalloproteinases (MMPs) [67,69], that could be potentially beneficial in counteracting cartilage hypertrophy and degradation but, also, a lower ECM protein content [69]. On the other hand, the higher production of TGF-β, chemokines and anti-inflammatory cytokines could make the wjMSCs’ secretome a perfect candidate to control the inflammatory process [67,68,69,70]. Similarly, EVs and, in particular, exosomes isolated from both wjMSCs and cbMSCs exerted antifibrotic [71,72], antiapoptotic, proliferative [32,73] and immunomodulatory effects [73,74,75] through the delivery to the target cells of several biological factors, including nucleic acids and proteins and, in particular, cytokines [32,73]. All this evidence suggests that secretome and EVs from ucMSCs have similar immunomodulatory and anti-inflammatory effects as compared to other MSCs types, although they present a different production of angiogenic factors and ECM proteins.

**Figure 1:**
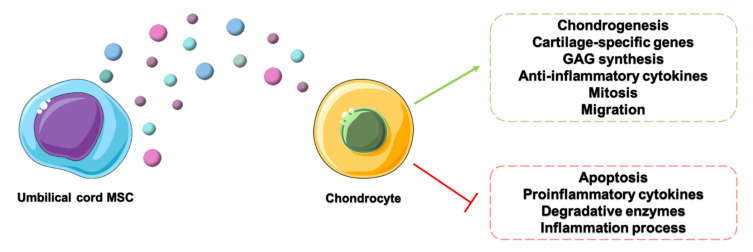
Effects of extracellular vesicles (EVs) from umbilical cord mesenchymal stem cells (ucMSCs) on osteoarthritis (OA) chondrocytes evidenced by in vitro studies. GAG: glycosaminoglycans.

This effect was found to be exerted in several different tissues [76,77], though few studies assessed the effects of ucMSCs in the musculoskeletal system. Instead, beneficial effects on arthritic diseases have been reported for molecules contained in EVs derived from MSCs isolated from other tissues. As an example, it has been demonstrated that long noncoding RNA KLF3-AS1 contained in bone marrow MSC-derived EVs inhibited miR-206, a miRNA that resulted in an overexpression in OA [16]. For instance, some papers reported that the secretome of cbMSCs can stimulate the chondrogenesis process [78] and that the secretome of wjMSCs can determine an increase in the expression of cartilage-specific genes in chondrocytes [59]. This evidence, combined with the previously described anti-inflammatory, antiangiogenic and proliferative effects, supports the potential beneficial effects of secretome and EVs of wjMSCs and cbMSCs, stimulating the regeneration process of articular tissues, as shown in Figure 1. More specifically, regarding joint arthritic diseases, Jeong et al. [60] demonstrated that cbMSCs did not directly differentiate towards a chondrocyte phenotype, but they exerted their actions through the secretion of paracrine factors. The secretome of cbMSCs treated with OA synovial fluid promoted the differentiation of chondroprogenitor cells in chondrocytes. The authors also reported that thrombospondin-2, a glycoprotein mediating cell-to-cell and cell-to-matrix interactions, was the key factor for this process, as it was able to activate in recipient cells different signaling pathways involved in chondrogenesis and cartilage regeneration. ucMSCs were also effective in the treatment of RA, especially when cultured in a 3D environment [61]. In fact, the secretome derived from the 3D culture of these cells, compared to their culture in a 2D environment, resulted richer in anti-inflammatory cytokines (such as IL-10 and LIF) and trophic factors (GM-CSF and many others) and stimulated both mitogenic features and glycosaminoglycan synthesis. This contributed to explaining the better outcomes found in an in vivo adjuvant-induced model of the disease obtained with the secretome from the 3D-cultured ucMSCs. Additionally, Yan and collaborators [62] reached similar conclusions, reporting that ucMSCs cultured in a 3D environment within a hollow-fiber bioreactor secreted a higher number of exosomes with respect to the same cells cultured in conventional 2D cultures. These EVs also stimulated the proliferation, migration and matrix synthesis, while decreasing the apoptosis rate, of in vitro arthritic chondrocytes. In accordance to the previous work, they also demonstrated that exosomes secreted by 3D-cultured ucMSCs had more regenerative potential in an in vivo model of a cartilage defect than those produced in a 2D environment. Despite the limited number of experimental evidences, secretome and EVs from ucMSCs have the potential to open a novel path in the treatment of arthritic joint diseases, possibly leading to the so-called “cell therapy without cells”.

## 5. Discussion

### 5.1. Open Clinical Challenges

Evidence from in vivo, in vitro and clinical data (summarized in Table 3) suggests a huge potential of umbilical cord-derived MSCs as a therapy for arthritic diseases. Although there seems to be a substantial consensus on their beneficial effects on diseased cartilage, there is still no clear evidence on the prevalent mechanism through which it is exerted. On one side, the traditional paradigm which bases cartilage healing on the MSCs homing and differentiation into chondrocytes is supported by the chondrogenic potential shown by cbMSCs and, even if more limited, by wjMSCs. To further boost cartilage formation by these cells, several approaches have been proposed, ranging from the supplementation of biochemical factors like IGF-1 [63], BMPs [79] or combinations [80] through the inhibition or overexpression of specific molecules [81,82,83] or the application of physical stimulation [84,85] and the coculture with other cell types [86].

More recently, however, an alternative mechanism at the basis of cartilage healing has gained attention, identifying in the paracrine secretions of MSCs the source of trophic factors able to induce articular tissue regeneration. In this context, the preliminary results obtained from the application of wjMSCs and cbMSCs’ secretome in arthritic settings, when compared to those obtained with the use of the whole cells, corroborate this view. A recent study comparing the use of amniotic fluid-derived MSCs to their exosomes in an animal model of OA shows superior cartilage regeneration and better pain tolerance in the exosome-treated animals as compared to cell-treated [87], further supporting the use of cell secretome as a therapeutic option.

From a clinical point of view, the possibility to use cell secretomes instead of cells themselves could represent a viable option, with routes of administration and procedures in-line with available treatments such as viscosupplementation and PRP injections. This approach could be considered even safer than classical surgical techniques. However, since biological factors contained in the secretome could have a short half-life in the articular environment, the effect of cell secretomes on cartilage regeneration would have a short-term effect, requiring repeated injections. On the contrary, a cell-based treatment in which cells can home and engraft in the articular niche and act as “secretome-factories” could assure a long-term and more robust effect. In both cases, for a standardized and routine clinical application, some critical aspects still need to be addressed. Firstly, the influence of a diseased environment on cell performance has to be taken into account. The ability of wjMSCs and cbMSCs to withstand inflammatory conditions without losing their biological activity is presently not yet unanimously acknowledged; in a study, wjMSCs showed a better retention of immunosuppressive ability when primed with TNF-α and IFN-γ, as compared to bmMSCs [88], while in a mouse model of OA, the presence of TNF-α inhibited cartilage restoration by human wjMSCs [89]. Secondly, dosing and timing are key factors to be considered in the design of an optimized therapeutic regimen based on umbilical cord-derived MSCs or on their EV injections. To this end, a recent study compared different injection schemes of wjMSCs (different numbers of injections and different doses), showing how multiple injections at higher doses decreased inflammatory signs and improved cartilage quality as compared to the other combinations [90]. Initial studies have been published also to establish an optimal dosage of EVs; in the area of graft-versus-host disease, the standardization of the effective dose was paramount to reach a therapeutic effect [91].

### 5.2. Open Industrial Challenges

From the industrial and pharmaceutical perspective, there are several aspects that can favor the use of MSC-derived EVs instead of the canonical cell therapy. Compared to a cellular product, isolated EVs could have advantages in terms of safety and ease of handling, thanks to their low toxicity, high stability, biocompatibility and biological barrier permeability. Moreover, EVs demonstrate a strong immunomodulatory potential. Thus, they can be conceived for both autologous and allogeneic applications. This would be particularly useful in a context of urgent treatment where the allogeneic use would allow the availability of off-the-shelf EV-based products ready for injections [92].

In accordance with the current regulatory guidelines regarding EVs, they can be classified as biological medicines and belong to the pharmaceutical class of biologicals. However, differently from other biopharmaceuticals such as recombinant proteins or antibodies, EVs are complex entities and may have much in common with their cell source. For this reason, the development of EV-based therapeutics should take into consideration not only the regulations for biologicals but, also, those for cell-based products and ATMPs, as described by Lener and collaborators [93]. In this regard, one of the most challenging aspects to be investigated is the definition of the “active substances” responsible of the biological effects and the “mode or mechanism of action” (MoA) of EVs. In this specific case, it is necessary to characterize the content of the vesicles and define the quality control strategy, including a series of quality and potency tests aimed to hypothesize the MoA [93]. To overcome this challenging task at the early stages of research on biological therapeutics (at the level of the investigational medicinal product), it is becoming common to rely on a “quality by design” approach, in which a strict control on the manufacturing process assures the quality of the obtained product, leaving the determination of the exact MoA and exploiting relevant in vivo and in vitro tests for later development, after completion of the phase II clinical trials [94]. In this context, to comply with the regulations in force, it is important to consider that the EVs must be produced according to the current good manufacturing practice (GMP) as a medicinal product [95]. This has to be applied not only to the final product itself (EVs) but, also, to the starting materials (the cells, MSCs) and the quality controls necessary to approve the final batch before release.

**Figure 2:**
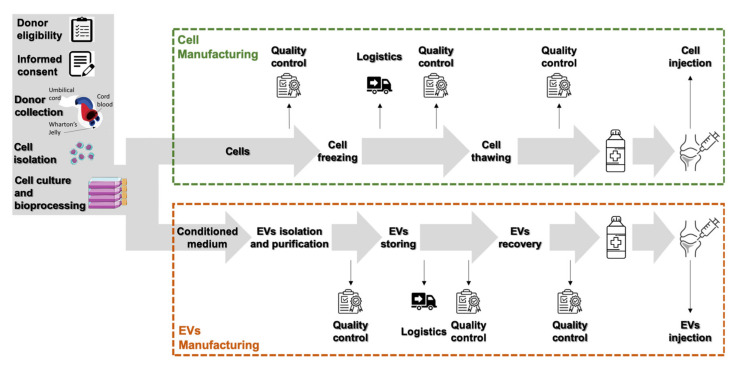
Manufacturing process for umbilical cord-derived MSCs and their EVs.

Indeed, the EVs’ manufacturing process would share a common starting point with the process of MSCs’ production, as depicted in Figure 2. Both cell and EV production processes would start with the selection of donors and the collection of informed consent and of the umbilical cord. Considering the much higher yield, wjMSCs represent a more viable option than cbMSCs for widespread clinical applications; thus, the process should progress with the enzymatic isolation of cells from Wharton’s jelly. From this point on, for the production of EVs, a master cell bank has to be established, from which specific cells lots are cultured to produce the conditioned medium for the subsequent EVs’ isolation, purification and storage. In case of cell therapy manufacturing, after isolation and characterization, cells would be expanded in the culture, stored and preserved until use.

The first aspect to consider in developing a process for EV manufacturing is the optimization of the methods used for the isolation and culture of wjMSCs. Cell culture conditions have to be standardized to assure the maintenance of wjMSC immunological profiles [96]. Cell culture parameters are crucial also for the production of EVs with the desired properties [97]. The composition of the EVs’ cargo, which in turn determines their biological effects on the target cells, depends on the conditions in which the MSCs are cultured [98]; indeed, as recently reviewed [92], both biophysical and biochemical signals can influence the cargo and the biological activities of the EVs secreted from MSCs. Furthermore, it has been shown that using MSCs at passages above the fourth affected the healing potential of their secreted EVs [99]. A possible solution allowing to scale-up the production of EVs resides in the use of immortalized EVs, since it has been demonstrated that EVs produced from immortalized EVs maintained the same therapeutic potential as compared to those derived from primary MSCs [100]. Finally, for a GMP-compliant process, it is crucial to overcome the main actual limits of EVs and cell production processes, such as the use of open systems during cells’ isolation, the availability of completely xeno-free reagents necessary for the expansion and the difficulty in the process step scale-up. To address these needs, it is imperative to develop a process as closed as possible, encompassing all the production steps and the full downstream phases (purification, concentration and final sterilization through filtration of the produced EVs). Some manufacturers are starting to use bioreactors [101], and in this case, it is important to consider that, beyond the amplification and purification steps, also the filtration and preparation of the final EV products must be carried out in a closed system.

Another crucial step in achieving a GMP-compliant EV production is the establishment of a complete quality control (QC) strategy suitable for the obtainment of a clinical trial authorization by the regulatory authority. The approval of the master cell bank, as well as of the single EVs lot (released for clinical use), is indeed based on passing a defined list of tests. Each test result should fall within limits (specifications) defined at a compendia level for parameters such as sterility, endotoxin level, mycoplasma and cell viability (European Pharmacopoeia). Alternatively, for other product characteristics, limits of acceptance should be set during the development phase, based on literature data and previous experimental results. An extensive safety panel, including adventitious viruses, karyotype, cell senescence and tumorigenicity, is mandatory for the master cell bank and the cultured cells used to obtain EVs from the conditioned medium.

To verify the sensitivity, precision, accuracy, reproducibility and robustness of the different production batches, in vitro tests need to be designed and validated according to regulatory guidelines (ICH International Council for Harmonisation of Technical Requirements for Pharmaceuticals for Human Use). For identity and purity evaluations, EVs’ quantity, size and surface markers profile, together with the total protein content and miRNA/RNA profile, can be determined.

Beside the safety and reliability of the production process, the efficacy of the product has to be tested by means of relevant in vitro and in vivo potency assays based on the target disease and the supposed MoA [102]. If available, the potency test needs to be validated according to GMP guidelines. In this context, in vitro assays to measure a specific biological activity may provide additional information, such as supplying data on the absence of proinflammatory cytokines, evaluating the antiapoptotic and the anti-inflammatory activity and, in the specific case of OA, the ability to accelerate cartilage repair. Finally, in vivo assays on OA animal models or on artificial constructs mimicking the cartilage/bone surface could complete the understanding on the MoA.

In the development of a cellular or GMP-EV therapeutic product, cryopreservation represents a key factor and a potential bottleneck, directly influencing the efficacy and stability of the medicinal product. In this process, there are several interrelated steps [103], but the most important for the purpose of this review are the choice of cryoprotective agents (CPAs), the storage and the distribution. The CPAs must be used in an optimal concentration to avoid, on one hand, the chilling shock (low concentration), but, on the other hand, also the potential toxicity related to too-high concentrations [104]. Considering the debate surrounding the use of dimethyl sulfoxide (DMSO), one of the most used CPAs, trehalose, is emerging as an ideal candidate among alternative CPAs. Its effectiveness has been reported both for stem cells [105] and for EVs [106]. Regarding the storage, there are important and crucial differences between cells and EVs. In fact, for cell therapies, the actual recommended practice is to maintain the preparations at temperatures below −135 °C [103]. This implies the choice between storage in liquid nitrogen (or better, in the gas phase above liquid nitrogen, at about −180 °C) or the employment of a mechanical refrigeration freezer able to reach these temperatures, each with their advantages and drawbacks [103]. Differently, an emerging number of studies demonstrated that frozen EVs are stable if stored at −80 °C for long periods [107], implying a lower cost for the equipment and its management. Moreover, in contrast to the cell products, EVs can be lyophilized or freeze-dried, increasing the EVs’ shelf life and reducing the storage demands and the costs associated with a simpler cold chain. Indeed, it has been demonstrated that the best storage temperature for lyophilized EVs is 4 °C [108]. In addition, the FDA already approved the clinical use of several preparations based on lyophilized liposomes [108,109], entities closely related to the EVs. These differences will be reflected also in the distribution process, during which the storage temperature must be maintained as constant as possible to assure the integrity and the stability of the products. In the case of cell preparations, there are essentially two methods to transport frozen cells: in liquid nitrogen (at temperatures of −180 °C or below) in appropriate and expensive carriers or in dry ice within insulated boxes (at a higher temperature of −80 °C). Regarding the EVs, the product could be shipped within the boxes filled with dry ice or, if lyophilized, at 4 °C, simplifying the logistic chain. Even if initial evidence started to demonstrate the maintenance of the biological activity of thawed EVs [110], further stability studies are necessary to assess that there is no loss of functional activity in long-term storage conditions [111].

## 6. Conclusions

In conclusion, umbilical cord-derived MSCs and their EVs represent a promising candidate for the therapy of OA, as highlighted by the encouraging results emerged from in vitro and in vivo investigations and from the preliminary clinical trials with cells. Even if EVs share with MSCs the same regulatory framework, the manufacturing of EVs could imply advantages compared to the use of cells in terms of easier handling, storage and distribution. Further research on the MoA of these biological therapeutics is needed and will help to foster the adoption of allogeneic, off-the-shelf cell therapies, with or without cells, in the field of arthritic diseases.

## Figures and Tables

**Table 1:** Details of the studies that used mesenchymal stem cells (MSCs) from the umbilical cord or their secretome/extracellular vesicles (EVs) in the treatment of in vitro models of joint diseases. Abbreviations: wjMSCs: Wharton’s jelly mesenchymal stem cells, OA: osteoarthritis, IL: interleukin, IGF: insulin-like growth factor, ADAMTS: a disintegrin-like and metalloproteinase with thrombospondin, MMP: matrix metalloproteinase, SOX: SRY-Box transcription factor, COX: cyclooxygenase, MIA: monosodium iodoacetate, PGE2: prostaglandin E, ucMSCs: umbilical cord mesenchymal stem cells, RA: rheumatoid arthritis, TIMP: tissue inhibitors of metalloproteinases, cbMSCs: cord blood mesenchymal stem cells, TNF-α: tumor necrosis factor-alpha, ICAM: intercellular adhesion molecule, TGF-β: transforming growth factor-beta, BMP: bone morphogenetic protein, COMP: cartilage oligomeric matrix protein, GAGs: glycosaminoglycans and Exo: exosomes. n.a.: not applicable.

References	Cell Types /Source	Secretome/Vesicle Type	Pathology	Target Cells	Culture System	Results
Widowati et al., 2018 [41]	Human wjMSCs	n.a.	OA	Human Chondrocyte cel lline + IL1β	Direct Co-culture	IGF1-1 ↑ wjMSCs chondrogenesis. Co-culture ↓ ADAMTS1, MMP1, MMP3
Wang et al., 2018 [42]	Human wjMSCs	Secretome (only for proliferation assay)	OA	Human Chondrocytes	Transwell Co-culture	wjMSCs secretome ↑ proliferation Co-culture ↑ aggrecan, Sox-9, collagen II, ↓ cox2, collagen X, MMP13, inflammatory factors
Chang et al., 2018 [45]	Human wjMSCs	Secretome	OA	Human Chondrocytes ± MIA	Indirect co-culture	wjMSCs secretome ↑ cell viability and ↓ apoptosis in damaged chondrocytes
Sofia et al., 2019 [46,47]	Human wjMSCs	n.a.	OA	Human synoviocytes	Direct Co-colture	Co-culture ↑ PGE2 and ↓ MMP13 and RELA
Zeng et al., 2016 [48]	Human ucMSCs	n.a.	RA	Human fibroblast-like synoviocytes	Co-culture	Co-culture ↑ synoviocyte apoptosis, aggrecan and collagen II, ↓ IL-1β, IL-6 and CCL-2
Saulnier et al., 2015 [52]	Equine wjMSCs	Secretome	OA	Rabbit IL1β-treated synoviocytes	Indirect co-culture	wjMSCs secretome ↓ MMP-1, -3, -13, IL1β, TIMP
Lo et al., 2013 [53]	Human cbMSCs	n.a.	OA	Human chondrocytes, treated with IL1β and TNFα	Direct Co-culture	Co-culture ↑ proliferation, integrins, ICAM-1, BMP-4, TGF-b1, SOX9, collagen 2, IL-6, IL-10 and aggrecan, ↓ cell death
Li et al., 2016 [59]	Human cbMSCs	Secretome	n.a.	Human articular chondrocytes	Direct and indirect co-cultures	Direct and indirect co-cultures ↑ SOX9, collagen II, TGFβ1, cell proliferation direct co-culture ↑collagen 2 and 1
Hassan Famian et al., 2017 [60]	Human wjMSCs	Secretome	Femoral neck fractures	Human chondrocytes	Monolayer or micromass culture	Secretome ↑ SOX9, collagen II, aggrecan and COMP, micromass > monolayer
Jeong et al., 2013 [61]	Human cbMSCs	Secretome	n.a.	Mouse chondroprogenitor cells from limb buds	Micromass culture	Secretome ↑ size of micromasses, lacunae number, collagen 2 and GAG
Miranda et al., 2019 [62]	Human ucMSCs	Secretome	n.a.	Mouse chondrocytes	ucMSCs 2D or 3D, monolayer chondrocytes	Secretome from 3D vs 2D ↑ anti-inflammatory and regenerative factors, chondrocyte migration, ↓ GAG synthesis
Yan et al., 2019 [63]	Human ucMSCs	Exosomes (average size: 120 nm)	OA	Human chondrocytes	ucMSCs cultured in 2D or 3D (hollow-fiber bioreactor), chondrocytes in 2D	3D-Exo ↑ cell proliferation, migration, collagen II, Sox9 and aggrecan, ↓ apoptosis, ADAMTS5, MMP13

**Table 2:** Details of the papers that used MSCs from the umbilical cord or their secretome/EVs in the treatment of joint diseases in in vivo models. Abbreviations: wjMSCs: Wharton’s jelly mesenchymal stem cells, OA: osteoarthritis, iNOS: inducible nitric oxide synthase, ADAMTS: a disintegrin-like and metalloproteinase with thrombospondin, cbMSCs: cord blood mesenchymal stem cells, RA: rheumatoid arthritis, HSC: hematopoietic stem cells, TNF-α: tumor necrosis factor-alpha, IL: interleukin, IFN-γ: interferon-gamma, GAGs: glycosaminoglycans, ICRS score: International Cartilage Repair Society score, MMP: matrix metalloproteinase, ucMSCs: umbilical cord mesenchymal stem cells and Exo: exosomes. n.a.: not applicable.

References	Cell Types/ Source	Secretome/Vesicle Type	Pathology	Study Design	Host	Results
Endrinaldi et al., 2019 [43]	wjMSCs	Secretome	OA (Chemically-induced)	Injection of wjMSCs (1 × 10^6^) vs secretome	Rats	Injection of wjMSCs ↑ serum level of iNOS while ADAMTS4 was = to secretome
Greish et al., 2012 [44]	Human cbMSCs	n.a.	RA (Chemically-induced)	Injection of cbMSCs or HSC (1 × 10^6^) twice a week for 5 weeks vs methotrexate	Rat	Injection of cbMSCs and HSC ↓ mean arthritis score, paw diameter, leucocyte infiltration, synovial hypertrophy than methotrexat; serum levels of TNF-α, IL-1 and IFN-γ did not significantly decrease
Chang et al., 2018 [45]	Human wjMSCs	n.a.	OA (Chemically-induced)	Injection of wjMSCs (1 × 10^5^)	Mouse	Injection of wjMSCs ↓ movement impairment and apoptosis; ↑ GAGs, collagen II, aggrecan and ICRS score
Zhang et al., 2018 [50]	Canine wjMSCs	n.a.	OA (Surgically-induced)	Injection of wjMSCs (1 × 10^6^ twice)	Dog (allogeneic)	Injection of wjMSCs ↑ recovery and cartilage thickness by imaging techniques; ↓ level of IL-6, IL-7 and TNF-α
Wu et al., 2019 [51]	Human wjMSCs	n.a.	Osteochondral defect (Surgically-induced)	Injection of wjMSCs (5 × 10^6^) in HA (4%)	Minipig	Injection of wjMSCs ↑ cartilaginous matrix, ICRS score and expression of chondrogenic markers; ↓ hypertrophic and catabolic markers
Saulnier et al., 2015 [52]	Equine wjMSCs	n.a.	OA (Surgically-induced)	Injection of wjMSCs (3,5 × 10^6^) after 3 or 15 days	Rabbit	Early injection ↓ visual score, cartilage fibrillation and levels of MMP-1, -3 and -13; Early injection did not modify number of osteophytes, synoviopathy and inflammatory infiltrate
Miranda et al., 2019 [62]	Human ucMSCs	n.a.	RA (Biologically-induced)	Injection of ucMSCs (17 × 10^6^ cultured in 2D or 3D)	Rat	3D supernatant ↓ weight loss, paw swelling, arthritic index, osteolysis than 2D ucMSCs secretome
Yan et al., 2019 [63]	Human ucMSCs	Exosomes	Cartilage defects (Surgically-induced)	Injection of Exo (1 × 10^10^/mL) from 2D or 3D cultured ucMSCs	Rabbit	Injection of Exo from 3D cultured ucMSCs ↑ gross aspect and thickness of the cartilage, ICRS and Wakitani score

**Table 3:** Details of the papers describing clinical trials with MSCs from the umbilical cord for the treatment of joint diseases. Abbreviations: cbMSCs: cord blood mesenchymal stem cells, HA: hyaluronic acid, IKDC: International Knee Documentation and Committee score, WOMAC: Western Ontario and McMaster Universities Osteoarthritis Index, GAGs: glycosaminoglycans, VAS: visual analog scale, OA: osteoarthritis, wjMSCs: Wharton’s jelly mesenchymal stem cells and MOCART: Magnetic Resonance Observation of Cartilage Repair Tissue.

References	Cell Types/ Source	Pathology	Delivery Mode	Study Design	Patient number	Results
Park et al., 2017 [55]	Human cbMSCs	Osteochondral defect	Injection of cbMSCs (5 × 10^6^/mL) in HA (4%)	Case report	1	Injection of cbMSCs ↑ IKDC, WOMAC, cartilage-like aspect, GAGs, Collagen type II; ↓ VAS and Collagen type I, no bone formation
Park et al., 2017 [56]	Human cbMSCs	OA	Injection of cbMSCs (1.15/1.25 × 10^7^ or 1.65/2 × 10^7^) in HA	Open-label, single-arm, phase I/II	7	Adverse effects: mild to moderate. antithyroglobulin antibody level: elevated. Injection of cbMSCs: ↑ IKDC and aspect of hyaline-like cartilage; ↓ VAS
Song et al., 2020 [57]	Human cbMSCs	OA	Injection of cbMSCs (7.5 × 10^6^) in HA (4%) – Commercial	Retrospective case series	128	Injection of cbMSCs ↑ IKDC and MOCART; ↓ VAS and WOMAC
Matas et al., 2019 [58]	Human wjMSCs (pooled from 3 donors)	OA	Injection of wjMSCs (20 × 10^6^) once or twice vs HA injection	Randomized double-blind, controlled phase I/II	29	Adverse effects: acute synovitis and mild to moderate symptomatic effusion. Injection of wjMSCs: ↓ WOMAC, VAS, pain and disability than HA, the injections led to better but not significantly outcomes
Dilogo et al., 2020 [64]	Human wjMSCs	OA	Injection of wjMSCs (10 × 10^6^) in 2 mL secretome + 2 mL HA	open-label, single arm, phase I/II	29	Injection of wjMSCs ↓ VAS in more sever patients and ↓ WOMAC in all patients; ↑ IKDC in more sever patients
